# ﻿Taxonomic notes on the genus *Yunohamella* Yoshida, 2007 (Araneae, Theridiidae) from China, with two new species

**DOI:** 10.3897/zookeys.1224.138987

**Published:** 2025-01-21

**Authors:** Rui Zhong, Yang Zhong, He Zhang, Jie Liu, Changyong Liu, Kuai Chen, Changhao Hu

**Affiliations:** 1 State Key Laboratory of Biocatalysis and Enzyme Engineering & Centre for Behavioural Ecology & Evolution, School of Life Sciences, Hubei University, Wuhan 430062, Hubei, China; 2 School of Nuclear Technology and Chemistry & Biology, Hubei University of Science and Technology, Xianning 437100, Hubei, China; 3 Hebei Province Sweet Potato Breeding and Application Technology Innovation Center, Xingtai 054001, Hebei, China; 4 Guo Shoujing Innovation College, Xingtai University, Xingtai 054001, Hebei, China; 5 Hubei Key Laboratory of Regional Development and Environmental Response, Faculty of Resources and Environmental Science, Hubei University, Wuhan 430062, Hubei, China; 6 Qizimeishan National Nature Reserve Administration, Xuan’en 445500, Hubei, China; 7 Hubei Broad Nature Technology Service Co., Ltd., Wuhan 430079, Hubei, China

**Keywords:** Biodiversity, comb-foot spiders, morphology, new combination, synonym, taxonomy

## Abstract

Four *Yunohamella* species are reported from Hubei Province, China, including two new species: *Y.gutenbergi* R. Zhong, J. Liu & Hu, **sp. nov.** (♂) and *Y.mohorovicici* R. Zhong, J. Liu & Hu, **sp. nov.** (♂). *Yunohamellajiugongensis* (Liu & Zhong, 2023), **comb. nov.** is transferred from the genus *Cryptachaea* Archer, 1946, and *Y.lyrica* (Walckenaer, 1841) is newly recorded from Hubei Province and is considered as a senior synonym of *Platnickinamneon* (Bösenberg & Strand, 1906).

## ﻿Introduction

*Yunohamella* Yoshida, 2007 is a small genus within the family Theridiidae Sundevall, 1833. To date, the genus contains eight described species (World Spider Catalog 2024), which are primarily distributed across the Eurasian continent, with four species recorded from China: *Y.gibbosa* Gao & Li, 2014, *Y.lyrica* (Walckenaer, 1841), *Y.serpatusa* (Guan & Zhu, 1993), and *Y.subadulta* (Bösenberg & Strand, 1906) (Zhu et al. 1993; Guan 2002; Yoshida 2007; Gao and Li 2014; Marusik and Logunov 2017; World Spider Catalog 2024). The first reported species of *Yunohamella* were originally classified in the genus *Theridion* Walckenaer, 1805. In 2001, Yoshida revised many species of *Theridion*. He described a new genus *Takayus* Yoshida, 2001 and designated two species groups in this genus, the *takayensis* group and the *yunohamensis* group, on the difference of the following characteristics: color of body, pattern on abdomen, scapus of epigynum, embolus and tegular apophysis of male palpus, and natural history. Yoshida (2007) later established the new genus *Yunohamella* based on the *Takayusyunohamensis* group earlier designated by him (Yoshida 2001) and transferred three species from *Takayus* to *Yunohamella*.

*Yunohamella* is placed in the subfamily Theridiinae Sundevall, 1833, and its phylogenetic placement shows it to be a sister group of the former *Achaearanea* Strand, 1929 sensu lato, which now is recognized as the genera *Achaearanea*, *Campanicola* Yoshida, 2015, *Cryptachaea* Archer, 1946, *Nihonhimea* Yoshida, 2016, and *Parasteatoda* Archer, 1946 (Liu et al. 2016).

The Qizimeishan National Nature Reserve, in southwestern Hubei, is characterized by a karst landscape. The reserve boasts rich forest vegetation and complex topography, providing ideal habitats that support the diversity and proliferation of the flora and fauna. Spiders of this nature reserve were surveyed from 2023 to 2024. In the current paper, we describe two new species of the genus *Yunohamella* from Qizimeishan National Nature Reserve and provide additional two taxonomic amendments.

## ﻿Materials and methods

The specimens examined in this study are deposited in the Centre for Behavioral Ecology and Evolution (**CBEE**), College of Life Sciences, Hubei University in Wuhan and School of Nuclear Technology and Chemistry and Biology, Hubei University of Science and Technology (**HUST**) in Xianning. Specimens were examined using an Olympus SZX7 stereo microscope. Photographs were taken with a Leica M205 C stereo microscope, and final multifocal images were produced with Helicon Focus v. 7.7.0. The male palp was examined and photographed after dissection. The epigyne was examined after being dissected from the spider’s body. The epigyne was removed and treated in a warmed 0.1 mg/ml Protease K solution before study. All morphological measurements were calculated using a Leica M205 C stereo microscope. Eye diameters were taken at the widest point. Legs measurements are given as total length (femur, patella, tibia, metatarsus, tarsus). The terminologies used in figure legends follow Agnarsson (2004) and Agnarsson et al. (2007). All measurements were in millimeters (mm).

Abbreviations: **ALE** = anterior lateral eye; **AME** = anterior median eye; **BH** = basal haematodocha; **C** = conductor; **CD** = copulatory duct; **CHd** = cymbial hood; **CO** = copulatory opening; **E** = embolus; **EB** = embolic base; **FD** = fertilization duct; **MA** = median apophysis; **PLE** = posterior lateral eye; **PME** = posterior median eye; **S** = spermatheca; **SD** = sperm duct; **ST** = subtegulum; **T** = tegulum; **TA** = tegular apophysis; **I**, **II**, **III**, **IV** = legs I–IV.

## ﻿Results

### ﻿Taxonomy

**Family Theridiidae Sundevall, 1833**

#### ﻿Genus Yunohamella

Taxon classificationAnimaliaAraneaeTheridiidae

Yoshida, 2007

4CA2B02C-9D8F-50C1-902B-FBD2EDD53D3D

##### Type species.

*Theridionyunohamense* Bösenberg & Strand, 1906 (= *Yunohamellayunohamensis*) from Japan.

##### Diagnosis.

Species of *Yunohamella* are similar to those of *Takayus* (compare Figs 2A–D, 4A–D, 5C–E, 6C, D, 7D–F, 8A, B, 9D–F; Gao and Li 2014: figs 107–109; Marusik and Logunov 2017: figs 37–39; Lee and Kim 2021: figs 3E–G, 4A,B with Zhu 1998: figs 80B–E, 83B–E, 93B–E, 94B–E, 108B–E, 114B–F, 115B–E, 116B–E, 117B–E, 118B–E, 119B–E, 120B–D, 125B, C) in having a large tegulum and a small median apophysis, a conductor conjugating with tegulum. However, *Yunohamella* can be distinguished from *Takayus* by the following: embolus thin; tegular apophysis distinct; and epigyne without a pointed scapus or with a blunt scapus (vs embolus broad, tegular apophysis invisible before expanded, epigyne with a pointed scapus in *Takayus*).

Species of *Yunohamella* can be distinguished from *Theridion* (compare Figs 2A–D, 4A–D, 5C–E, 6C, D, 7D–F, 8A, B, 9D–F; Gao and Li 2014: figs 107–109; Marusik and Logunov 2017: figs 37–39; Lee and Kim 2021: figs 3E–G, 4A, B with Zhu 1998: figs 73B–E, 76B–E, 85B–E, 88B–E, 89B–D, 90B–E, 91B, C, 97B–E, 98B–E, 106B–E, 109B–E, 110B–D 123B–E, 124B–E) by the following: embolus short and straight; tegulum large; conductor conjugating with tegulum; epigyne without depression (vs embolus long and circular; tegulum not large; conductor separated; epigyne with a distinct depression in *Takayus*) (Yoshida 2007).

Species of *Yunohamella* can be distinguished from *Cryptachaea* (compare Figs 2D, 4D, 8A, B; Levi 1957: fig. 323 with Levi 1955: fig. 82) in having a median apophysis separated from the embolus and the present tegular apophysis (vs median apophysis attached to the embolus and tegular apophysis absent in *Cryptachaea*).

##### Distribution.

Asia, Europe, North America.

#### ﻿ Yunohamella gutenbergi

Taxon classificationAnimaliaAraneaeTheridiidae

R. Zhong, J. Liu & Hu sp. nov.

B516E6D0-3FDD-5DA5-98F2-C63F871DDA42

https://zoobank.org/C32395E0-14D0-4BD1-8E70-56C4D298B434

Figs 1 , 2 , 10

##### Type material.

***Holotype*** • male: China, Hubei Province: Enshi Tujia and Miao Autonomous Prefecture, Xuan’en County, Qizimeishan National Nature Reserve, Changtanhe Dong Autonomous Town, Shanyangxi; 30.08°N, 109.75°E; elev. 810 m; 3 July 2023; Changhao Hu & Mian Wei leg. (CBEE, QZMS01049).

##### Etymology.

The species is named after the geophysicist “Beno Gutenberg” who found the “core-mantle discontinuity”, the boundary between the mantle and the core of Earth.

##### Diagnosis.

Males of *Y.gutenbergi* R. Zhong, J. Liu & Hu, sp. nov. can be distinguished from all congeners in having a unique 2-shaped curved sperm duct on the tegulum and a filiform, curved embolus (Fig. 2A–C). Females are unknown.

##### Description.

**Male** (holotype) measurements: total length 2.37. Carapace 1.10 long, 0.94 wide. Abdomen 1.29 long, 0.90 wide. Eyes: AME 0.10, ALE 0.08, PME 0.07, PLE 0.08, AME–AME 0.07, AME–ALE 0.04, PME–PME 0.06, PME–PLE 0.08, AME–PME 0.10, ALE–PLE 0.00. Measurements of legs [leg II missing]: I — (2.98, —, —, —, —), III 4.09 (1.38, 0.30, 0.86, 1.05, 0.50), IV 5.12 (1.79, 0.32, 1.35, 1.36, 0.30).

**Figure 1.**
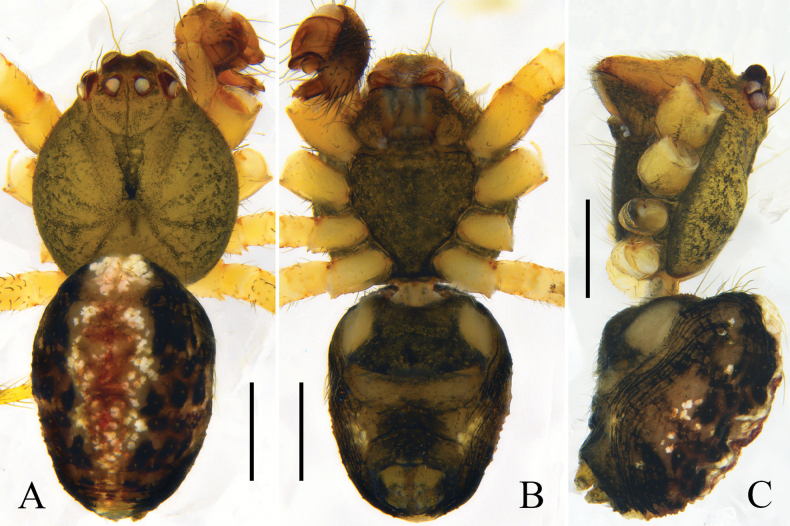
*Yunohamellagutenbergi* R. Zhong, J. Liu & Hu, sp. nov., male habitus. **A** dorsal view **B** ventral view **C** lateral view. Scale bars: 0.5 mm. (photos by Changhao Hu and Rui Zhong.)

Carapace round, brown, and with a narrow, trapezoid, black mark between head region and median furrow; radial furrow black. Sternum shaped like an inverted triangle and brown. Chelicerae and legs orange. Abdomen oval; dorsally black, with a longitudinal mark composed of white and red spots; venter dark brown; anterior part of genital groove and anterior part of spinnerets black; lateral abdomen with several white spots. Spinnerets dark brown (Fig. 1A–C).

**Figure 2.**
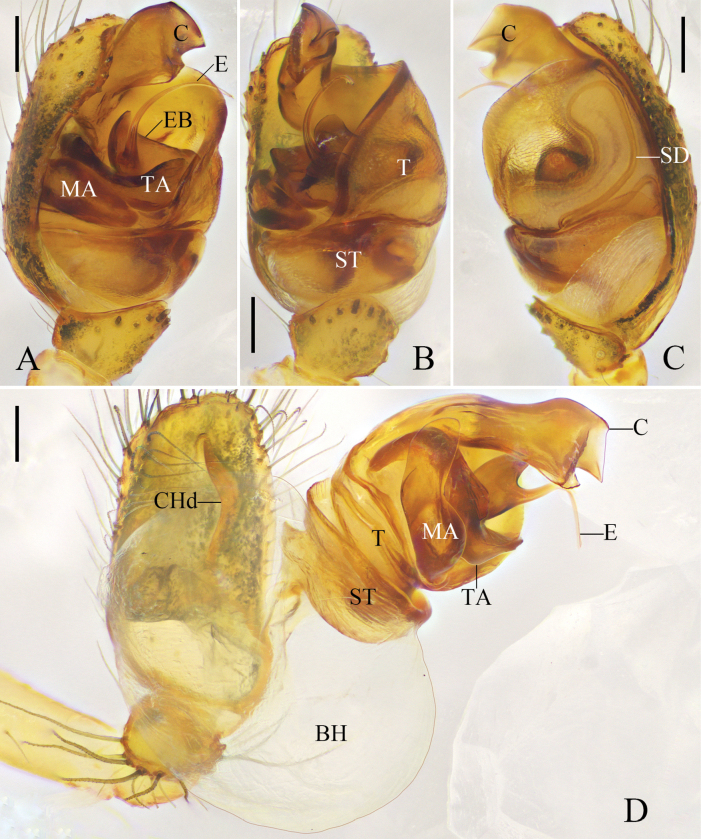
*Yunohamellagutenbergi* R. Zhong, J. Liu & Hu, sp. nov., left male palp. **A** prolateral view **B** ventral view **C** retrolateral view **D** expanded, ventral view. Scale bars: 0.2 mm. (Photos by Changhao Hu.)

Cymbium reniform. Cymbial hood longitudinal, almost ½ length of cymbium. Subtegulum bowl-shaped. Tegulum with a narrow prolateral part and a large retrolateral part; retrolateral part with a thin area that holds embolic base; sperm duct half surrounds thin area, and extends as 2-shaped, then straight down. Median apophysis lamellar. Tegular apophysis irregular. Length of median apophysis and tegular apophysis almost as long as width of bulb. Conductor sclerotized, with a triangular terminal apophysis. Embolus filiform and curved, with a lamellar base (Fig. 2A–D).

**Female.** Unknown.

##### Distribution.

Known only from the type locality (Fig. 10).

#### ﻿ Yunohamella mohorovicici

Taxon classificationAnimaliaAraneaeTheridiidae

R. Zhong, J. Liu & Hu sp. nov.

B275CA0B-3CBA-5DC6-A238-CD928B24778C

https://zoobank.org/8F92051B-B279-4121-AB6A-F37AD1FE3B27

Figs 3 , 4 , 10

##### Type material.

***Holotype*** • male: China, Hubei Province: Enshi Tujia and Miao Autonomous Prefecture, Xuan’en County, Qizimeishan National Nature Reserve, Changtanhe Dong Autonomous Town, Qizimeishan mountain; 30.03°N, 109.73°E; elev. 1270 m; 6 July 2023; Changhao Hu & Mian Wei leg. (CBEE, QZMS04642).

##### Etymology.

The species is named after the geophysicist “Andrija Mohorovičić” who found the “Moho discontinuity”, the boundary between the crust and the mantle of Earth.

##### Diagnosis.

Males of *Y.mohorovicici* sp. nov. are similar to those of *Y.jiugongensis* (Liu & Zhong, 2023) comb. nov. (compare Fig. 4A–C with Fig. 5C–E) in having an n-shaped sperm duct on the tegulum and a thick, curved embolus, but *Y.mohorovicici* can be distinguished from *Y.jiugongensis* by the following: sharp tooth-like apophysis on tegulum absent; and terminal conductor rounded (vs apophysis on tegulum present and terminal conductor knife-shaped in *Y.jiugongensis* comb. nov.). Males of *Y.mohorovicici* sp. nov. are also similar to those of *Y.palmgreni* (Marusik & Tsellarius, 1986) (compare Fig. 4A–C with Marusik and Tsellarius 1986: figs 1, 2) in having an n-shaped sperm duct on the tegulum and a thick embolus, but *Y.mohorovicici* can be distinguished from *Y.palmgreni* by the following: conductor arising from the retrolateral part of the tegulum at the 2 o’clock position; and embolus curved (vs conductor arising from retrolateral part of the tegulum at the 12 o’clock position and embolus straight in *Y.palmgreni*). Females are unknown.

##### Description.

**Male** (holotype), measurements: total length 1.77. Carapace 0.96 long, 0.77 wide. Abdomen 0.88 long, 0.65 wide. Eyes: AME 0.11, ALE 0.08, PME 0.07, PLE 0.07, AME–AME 0.06, AME–ALE 0.03, PME–PME 0.05, PME–PLE 0.08, AME–PME 0.05, ALE–PLE 0.01. Measurements of legs: I 6.80 (2.05, 0.37, 1.78, 1.92, 0.68), II 4.15 (1.29, 0.27, 0.99, 1.09, 0.51), III 2.70 (0.89, 0.19, 0.55, 0.67, 0.40), IV 3.66 (1.21, 0.25, 0.81, 0.95, 0.44). Leg formula: I-II-IV-III.

**Figure 3.**
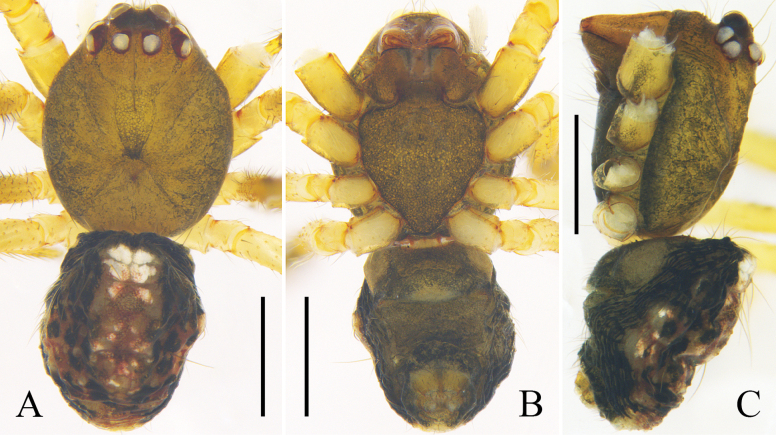
*Yunohamellamohorovicici* R. Zhong, J. Liu & Hu, sp. nov., male habitus. **A** dorsal view **B** ventral view **C** lateral view. Scale bars: 0.5 mm. (Photos by Changhao Hu and Rui Zhong.)

Carapace round, brown, with deep fovea and black radial furrow. Sternum shaped like an inverted triangle and brown. Labium brown. Chelicerae and endites orange. Legs yellow. Abdomen oval, with long hairs; dorsum black, with a longitudinal mark composed of white and red spots; venter brownish green; anterior part of spinnerets black; lateral abdomen with several white spots. Spinnerets brown (Fig. 3A–C).

**Figure 4.**
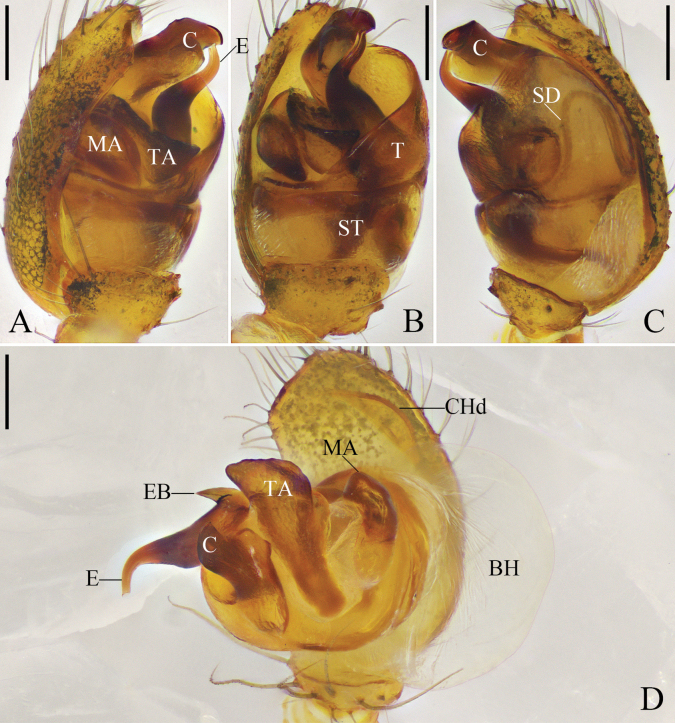
*Yunohamellamohorovicici* R. Zhong, J. Liu & Hu, sp. nov., left male palp. **A** prolateral view **B** ventral view **C** retrolateral view **D** expanded, ventral view. Scale bars: 0.2 mm. (Photos by Changhao Hu.)

**Figure 5.**
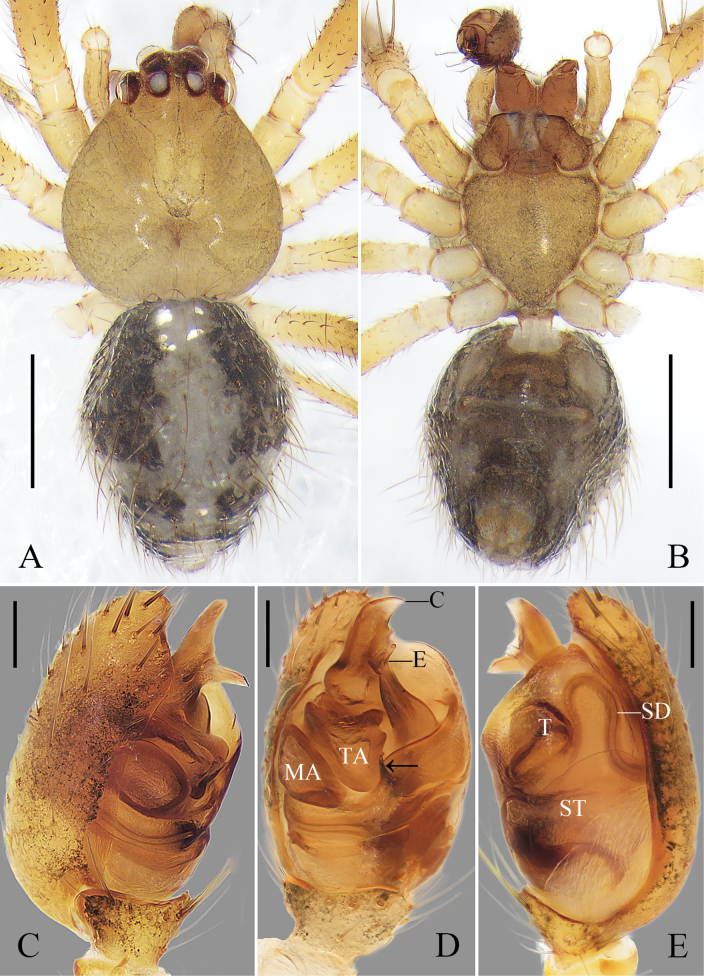
*Yunohamellajiugongensis* (Liu & Zhong, 2023) comb. nov., male. **A** habitus, dorsal view **B** habitus, ventral view **C** left palp, prolateral view **D** left palp, ventral view (arrow points to the sharp tooth-like apophysis on tegulum) **E** left palp, retrolateral view. Scale bars: 0.5 mm (**A, B**); 0.1 mm (**C–E**). (Photos by Yang Zhong.)

Cymbium reniform. Cymbial hood tilted at 60°, almost ¼ length of cymbium. Subtegulum bowl-shaped. Tegulum with a narrow prolateral part and a large retrolateral part; retrolateral part with a thin area that holds embolic base; sperm duct narrowly n-shaped. Median apophysis small, almost ½ length of tegular apophysis. Tegular apophysis large, knife-shaped; length of tegular apophysis almost as long as width of bulb. Conductor sclerotized, with smooth end. Embolus corn-like, thick, and curved, with a tooth-shaped base (Fig. 4A–D).

**Female.** Unknown.

##### Distribution.

Known only from the type locality (Fig. 10).

#### ﻿ Yunohamella jiugongensis

Taxon classificationAnimaliaAraneaeTheridiidae

(Liu & Zhong, 2023) comb. nov.

03DF88AD-1C53-560A-A686-5A5E65F05E7E

Figs 5 , 6 , 10

Cryptachaea jiugongensis Liu and Zhong in Liu et al. 2023: 98, figs 1A–F, 2A–E.

##### Type material

(examined). ***Holotype*** • male: China, Hubei Province: Xianning City, Jiugongshan National Nature Reserve, Yunzhonghu scenic spot; 29.39°N, 114.65°E; elev. 1230 m; 27 June 2021; Yang Zhong, Feng Lu, Han Dong & Jiangwei Zheng leg. (HUST, ZY2024001). ***Paratypes*** • 2 females, same data as holotype (HUST, ZY2024002).

##### Diagnosis.

**Figure 6.**
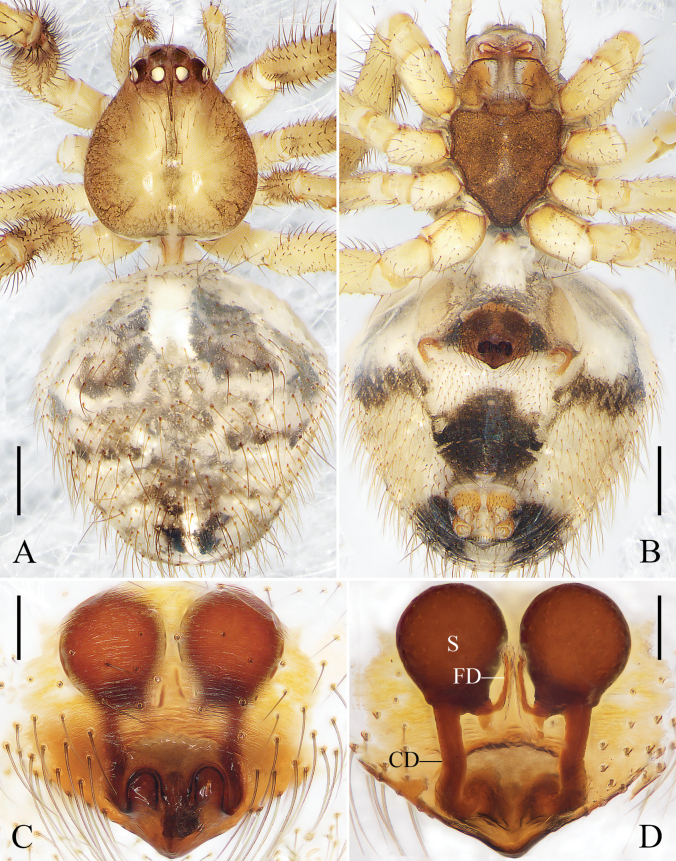
*Yunohamellajiugongensis* (Liu & Zhong, 2023) comb. nov., female. **A** habitus, dorsal view **B** habitus, ventral view **C** epigyne, ventral view **D** vulva, dorsal view. Scale bars: 0.5 mm (**A, B**); 0.1 mm (**C, D**). (Photos by Yang Zhong.)

For males, see the above diagnosis under *Y.mohorovicici* sp. nov. Males of *Y.jiugongensis* comb. nov. are also similar to those of *Y.palmgreni* (compare Fig. 5C–E with Marusik and Tsellarius 1986: figs 1, 2) in having an n-shaped sperm duct on the tegulum and a thick embolus, but *Y.jiugongensis* comb. nov. can be distinguished from *Y.palmgreni* by the following: conductor arising from the retrolateral part of the tegulum at the 2 o’clock position; and embolus curved (vs conductor arising from the retrolateral part of tegulum at the 12 o’clock position and embolus straight in *Y.palmgreni*). Females of *Y.jiugongensis* comb. nov. are similar to those of *Y.serpatusa* (Guan & Zhu, 1993) (compare Fig. 6C, D with Esyunin and Efimik 1996: figs 3–5) in having a blunt scapus and the copulatory ducts almost as long as the diameter of the spermathecae, but *Y.jiugongensis* comb. nov. can be distinguished from *Y.serpatusa* by the parallel copulatory ducts (vs not parallel in *Y.serpatusa*). Females of *Y.jiugongensis* comb. nov. are also similar to those of *Y.yunohamensis* (Bösenberg & Strand, 1906) (compare Fig. 6C–D with Yoshida 2003: figs 233, 234) in having a scapus and copulatory ducts almost as long as the diameter of the spermathecae, but *Y.jiugongensis* comb. nov. can be distinguished from *Y.yunohamensis* by having an obtuse scapus and straight, parallel copulatory ducts (vs scapus with two rounded ends and copulatory ducts curved and not parallel in *Y.yunohamensis*).

##### Redescription.

Male, measurements: total length 1.77. Carapace 0.88 long, 0.76 wide. Abdomen 0.89 long, 0.77 wide. Eyes: AME 0.10, ALE 0.06, PME 0.08, PLE 0.07, AME–AME 0.06, AME–ALE 0.03, PME–PME 0.06, PME–PLE 0.02, ALE–PLE 0.02. Measurements of legs [legs I and IV missing]: II 3.36 (0.86, 0.30, 0.79, 0.93, 0.48), III 2.14 (0.67, 0.20, 0.38, 0.55, 0.34).

Carapace light brown, with black radial furrow. Sternum shaped like an inverted triangle and light brown. Chelicerae, labium, and endites brown. Legs yellow to orange. Abdomen black, with long hairs; dorsum with a grey longitudinal mark and some white spots; posterior dorsum with grey transverse marks; median band of ventral abdomen darker than the other bands. Spinnerets orange (Fig. 5A, B).

Subtegulum bowl-shaped. Tegulum with a narrow prolateral part and a large retrolateral part; retrolateral part with a thin area that holds embolic base; sperm duct narrowly n-shaped; tegulum with a sharp, tooth-like apophysis in ventral view. Median apophysis and tegular apophysis triangular in ventral view. Conductor sclerotized, with knife-shaped end. Embolus corn-like, thick, and curved (Fig. 5C–E).

Female, measurements: total length 3.53. Carapace 1.32 long, 1.16 wide. Abdomen 2.21 long, 1.81 wide. Eyes: AME 0.11, ALE 0.11, PME 0.09, PLE 0.12, AME–AME 0.07, AME–ALE 0.06, PME–PME 0.07, PME–PLE 0.06, ALE–PLE 0.02. Measurements of legs: I 7.90 (2.30, 0.42, 2.31, 2.09, 0.78), II 4.85 (1.45, 0.38, 1.22, 1.23, 0.57), III 3.08 (0.87, 0.26, 0.75, 0.66, 0.54), IV 5.56 (1.56, 0.53, 1.29, 1.51, 0.67). Leg formula: I-IV-II-III.

Carapace brown, with median band of carapace lighter than the rest of the carapace. Sternum brown. Abdomen light grey; dorsum with irregular black marks; posterior-lateral part of genital groove with black transverse bands, a rounded and two triangular black marks located around spinnerets. Other characters of habitus as for male (Fig. 6A, B).

Epigyne with a blunt scape; copulatory openings located medially at scape. Copulatory ducts straight and parallel, almost as long as diameter of spermathecae. Spermathecae spherical. Fertilization ducts thin, long, almost ½ diameter of spermathecae, and arising posteriorly from spermathecae (Fig. 6C, D).

##### Natural history.

This species inhabits bushes.

##### Comments.

The justification for the removal of *Y.jiugongensis* comb. nov. from *Cryptachaea* is supported by its distinct differences from diagnostic characteristics for *Cryptachaea*, particularly the median apophysis attached to the embolus and the absence of a tegular apophysis, a defining character for the genus *Cryptachaea* (Yoshida 2008; Rodrigues and Poeta 2015). In contrast, the male palp of *Y.jiugongensis* comb. nov. exhibits the presence of tegular apophysis. The species is placed into *Yunohamella* based on similarities of the palpal structures, specifically conductor conjugated with a large tegulum (Fig. 5C–E). This species also shares similarities in the epigynal structures with *Yunohamella*, specifically the presence of a blunt scapus (Fig. 6C, D), as well as similarities in the dark abdomen (Figs 5A, B, 6A, B). Consequently, we transfer *C.jiugongensis* from *Cryptachaea* to *Yunohamella* as a new combination.

##### Distribution.

Known only from the type locality (Fig. 10).

#### ﻿ Yunohamella lyrica

Taxon classificationAnimaliaAraneaeTheridiidae

(Walckenaer, 1841)

04FE2A05-1C73-5331-899C-DABEDE93DFB5

Figs 7 , 8 , 9 , 10

Theridion lyricum Walckenaer, 1841: 288; Archer 1946: 43; Levi 1957: 89, figs 322–323, 329–331; Yoshida 1987: 13, figs 1, 2; Yoshida 1989: 318, fig. 4P–R; Chikuni 1989: 44, fig. 64; Kim and Kim 2001: 155, fig. 2A–I; Namkung 2002: 96, fig. 13.14a, b; Paquin and Dupérré 2003: 223, figs 2494–2496.
Theridion lyra Hentz, 1850: 279, pl. 9, fig. 21; Keyserling 1884: 50, pl. 2, fig. 28; Kaston 1948: 106, figs 132, 153, 154.
Theridion kentuckyense Keyserling, 1884: 78, pl. 4, fig. 47; Banks 1892: 30, pl. 5, fig. 43; Emerton 1909: 180, pl. 1, fig. 6.
Theridion mneon Bösenberg & Strand, 1906: 142, pl. 12, fig. 286.
Allotheridion lyricum : Archer 1950: 20.
Takayus lyricus : Yoshida 2001: 167; Namkung 2003: 98, fig. 13.14a, b; Yoshida 2003: 97, figs 239–242, 530.
Keijia mneon : Yoshida 2001: 172.
Yunohamella lyrica Yoshida 2007: 69; Yoshida 2009: 372, figs 149, 150; Lee and Kim 2021: 166, fig. 4A; Kim 2021: 172, fig. 75A–D.
Platnickina mneon : Koçak and Kemal 2008: 3; Ono 2011: 452; Dupérré 2023: 231, fig. 73A–C. Syn. nov.

##### Material examined.

• 2 males, 4 females: China Hubei Province: Enshi Tujia and Miao Autonomous Prefecture, Xuan’en County, Qizimeishan National Nature Reserve, Chunmuying Town, Xiaoshui Cave; 30.02°N, 109.78°E; elev. 1777 m; 1 June 2024; Changhao Hu & Mian Wei leg. (CBEE, QZMS04713, QZMS04714, QZMS04751–QZMS04754). • 2 females: Enshi Tujia and Miao Autonomous Prefecture, Xuan’en County, Qizimeishan National Nature Reserve, Chunmuying Town, Xiaoshui Cave; 30.02°N, 109.78°E; elev. 1777 m; 12 July 2023; Changhao Hu & Mian Wei leg. (CBEE, QZMS02405, QZMS02406). • 1 female: Enshi Tujia and Miao Autonomous Prefecture, Xuan’en County, Qizimeishan National Nature Reserve, Chunmuying Town, Shaiping Village; 29.96°N, 109.76°E; elev. 1822 m; 31 July 2023; Changhao Hu & Mian Wei leg. (CBEE, QZMS01160).

##### Diagnosis.

**Figure 7.**
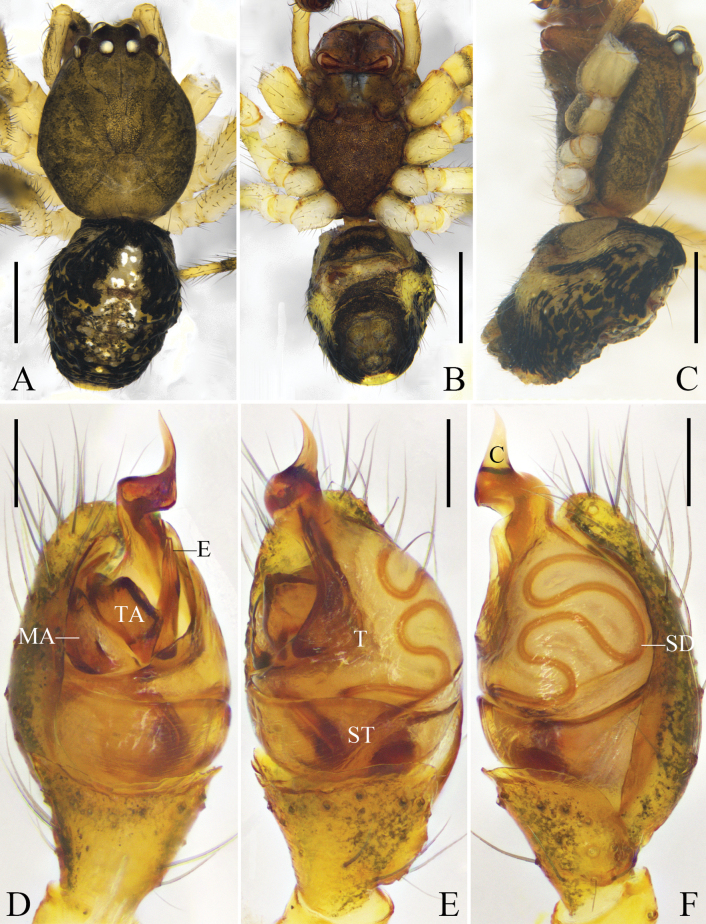
*Yunohamellalyrica* (Walckenaer, 1841), male. **A** habitus, dorsal view **B** habitus, ventral view **C** habitus, lateral view **D** left palp, prolateral view **E** left palp, ventral view **F** left palp, retrolateral view. Scale bars: 0.5 mm (**A–C**); 0.2 mm (**D–F**). (Photos by Changhao Hu and Rui Zhong.)

For males see the diagnosis under *Y.varietas* by Lee and Kim (2021). Males of *Y.lyrica* are also similar to those of *Y.subadulta* (compare Fig. 7D–F with Kim 2021: fig. 77E–G) in having a sharp terminal conductor and a sperm duct curving four times, but *Y.lyrica* can be distinguished from *Y.subadulta* in having the conductor extend beyond the cymbium (vs. conductor not exceeding the cymbium in *Y.palmgreni*). Females of *Y.lyrica* are similar to those of *Y.takasukai* Yoshida, 2012 (compare Fig. 9D–F with Yoshida 2012: figs 4, 5) in having a pair of projections on the anterlateral epigynal plate, but *Y.lyrica* can be distinguished from *Y.takasukai* by having oval, laminar projections on the anterlateral epigynal plate and long, curved copulatory ducts (vs nipple-like projections and copulatory ducts short in *Y.takasukai*).

##### Redescription.

Male, measurements: total length 2.11. Carapace 1.16 long, 0.88 wide. Abdomen 1.10 long, 0.91 wide. Eyes: AME 0.10, ALE 0.08, PME 0.08, PLE 0.08, AME–AME 0.10, AME–ALE 0.04, PME–PME 0.07, PME–PLE 0.09, AME–PME 0.09, ALE–PLE 0.00. Measurements of legs: I 7.48 (2.27, 0.43, 1.94, 2.10, 0.74), II 4.29 (1.38, 0.30, 0.98, 1.17, 0.46), III 2.71 (1.00, 0.19, 0.52, 0.60, 0.40), IV 3.87 (1.31, 0.26, 0.88, 0.95, 0.47). Leg formula: I-II-IV-III.

Carapace brownish green, with deep fovea and black radial furrow. Sternum shaped like an inverted triangle and dark brown. Chelicerae orange. Labium and endites dark brown. Legs yellow. Abdomen with long hairs; dorsum black, with a longitudinal mark composed of yellow base and white spots; venter yellow; anterior part of spinnerets black. Spinnerets brown (Fig. 7A–C).

**Figure 8.**
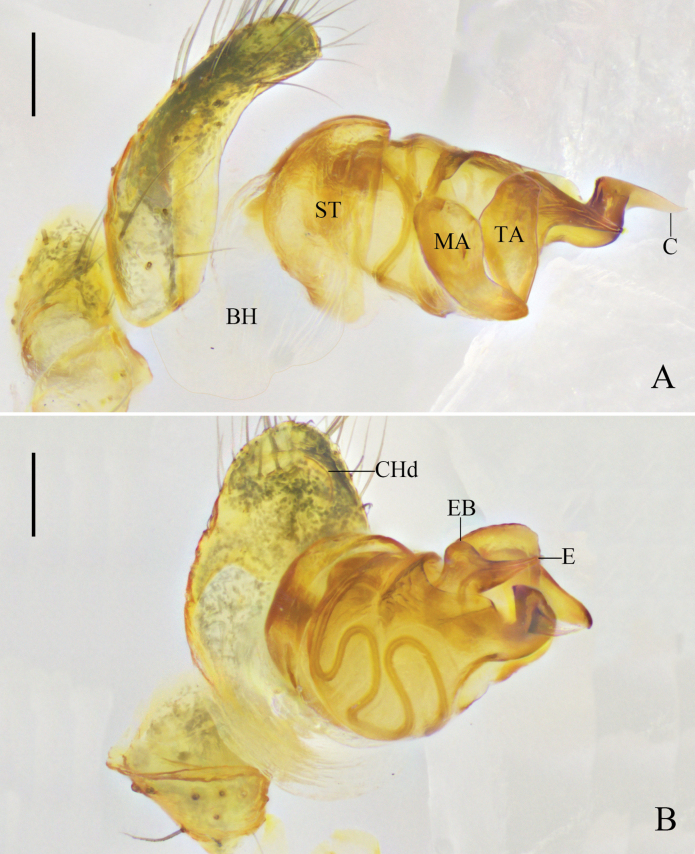
*Yunohamellalyrica* (Walckenaer, 1841), expanded left male palp. **A** prolateral view **B** ventral view. Scale bars: 0.2 mm. (Photos by Changhao Hu.)

Cymbium oval. Cymbial hood tilted at 30°, almost 1/8 length of cymbium. Subtegulum bowl-shaped. Tegulum with a narrow prolateral part and a large retrolateral part; sperm duct curving four times. Median apophysis and tegular apophysis almost as long as the width of bulb. Conductor sclerotized, with a sharp end. Embolus straight, with a rounded base (Figs 7D–F, 8A, B).

Female, measurements: total length 2.49. Carapace 0.91 long, 0.74 wide. Abdomen 1.40 long, 1.31 wide. Eyes: AME 0.09, ALE 0.07, PME 0.09, PLE 0.09, AME–AME 0.04, AME–ALE 0.02, PME–PME 0.08, PME–PLE 0.07, AME–PME 0.05, ALE–PLE 0.00. Measurements of legs: I 5.57 (1.73, 0.32, 1.47, 1.43, 0.62), II 3.76 (1.24, 0.33, 0.83, 0.86, 0.50), III 2.71 (0.94, 0.25, 0.51, 0.61, 0.40), IV 3.84 (1.30, 0.34, 0.84, 0.91, 0.45). Leg formula: I-IV-II-III.

**Figure 9.**
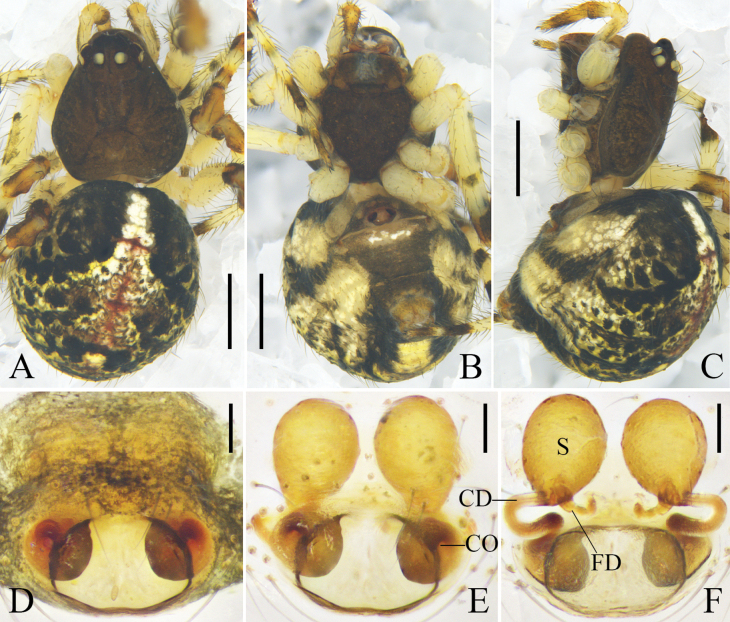
*Yunohamellalyrica* (Walckenaer, 1841), female. **A** habitus, dorsal view **B** habitus, ventral view **C** habitus, lateral view **D** uncleared epigyne, ventral view **E** uncleared epigyne, ventral view **F** vulva, dorsal view. Scale bars: 0.5 mm (**A–C**); 0.1 mm (**D–F**). (Photos by Changhao Hu.)

Carapace dark brown. Legs yellow to orange. Abdomen black; dorsum with a longitudinal mark composed of white, red, and yellow spots; posterior part of dorsum with three inverted V-shaped marks composed of yellow spots; median band of venter black, the rest yellow; posterior part of genital groove with several white spots, posterior-lateral part of genital groove with black transverse bands. Other characters of habitus as for male (Fig. 9A–C).

Epigyne with an oval atrium, two oval sclerotized plates overhanging anterolateral epigynal plate, copulatory openings located laterally on sclerotized plates. Copulatory ducts curved into a C-shape. Spermathecae spherical. Fertilization ducts arising posteriorly from spermathecae (Fig. 9D–F).

##### Natural history.

This is one of the most widespread *Yunohamella* species. This species is found on bushes, forests, and fences, as well as inside houses (Kaston 1948; Levi 1957; Gao and Li 2014; Kim et al. 2016; Lee and Kim 2021).

##### Comments.

*Platnickinamneon* was first described by Bösenberg and Strand (1906) based on a female specimen collected in Saga, Japan. To date, males of *P.mneon* remain unknown. Dupérré (2023) provided diagnostic characters and illustrations of *P.mneon* after examining the female holotype and noted that this species does not conform to the characteristics of the genus *Platnickina* Koçak & Kemal, 2008 (e.g. copulatory openings located on sclerotized plates in *P.mneon* vs inside the circular depression of the epigyne in *Platnickina* spp.) and that it likely belongs to another genus. The holotype of *P.mneon* exhibits all the diagnostic features of *Y.lyrica* as provided by Levi (1957), including the two sclerotized plates, copulatory openings located on the sclerotized plates, and oval spermathecae (compare Figs 7D–F, 9D–F with Dupérré 2023: fig. 73B, C and with Levi 1957: figs 329, 330). Although the types of *Y.lyrica* and *P.mneon* were unavailable for examination, our comparison based on specimens collected in Hubei Province, China and the illustrations and descriptions provided by Levi (1957) and Dupérré (2023) allows us to consider *P.mneon* as a junior synonym of *Y.lyrica*.

**Figure 10.**
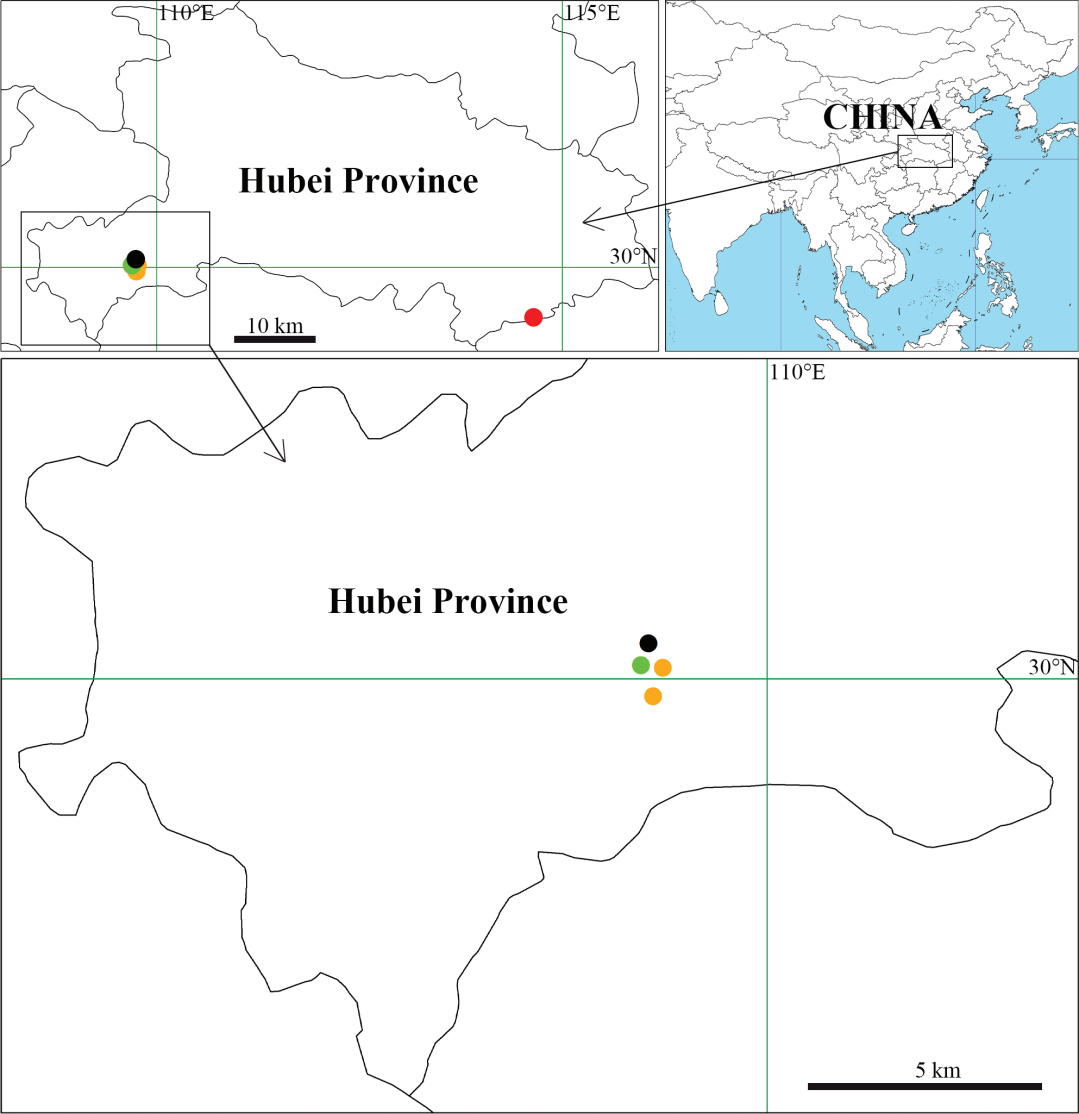
The collection locality of *Yunohamella* spp. from Hubei Province, China. Black circle represents *Y.gutenbergi* R. Zhong, J. Liu & Hu, sp. nov.; green circle represents *Y.mohorovicici* R. Zhong, J. Liu & Hu, sp. nov.; red circle represents *Y.jiugongensis* (Liu & Zhong, 2023) comb. nov.; orange circles represent *Y.lyrica* (Walckenaer, 1841).

##### Distribution.

China (Hubei Province, new Province record; Yunnan Province), Japan, Korea, North America.

## Supplementary Material

XML Treatment for Yunohamella

XML Treatment for Yunohamella gutenbergi

XML Treatment for Yunohamella mohorovicici

XML Treatment for Yunohamella jiugongensis

XML Treatment for Yunohamella lyrica

## ﻿Additional information

### Author ORCIDs

Rui Zhong https://orcid.org/0000-0001-5910-7302

Yang Zhong https://orcid.org/0000-0002-0517-4582

He Zhang https://orcid.org/0000-0002-1478-9837

Jie Liu https://orcid.org/0000-0001-7744-9744

Changhao Hu https://orcid.org/0009-0007-5591-3121

### Data availability

All of the data that support the findings of this study are available in the main text.
